# New Publications

**Published:** 1898-11

**Authors:** 


### NEW PUBLCATIONS.

The work of R. A. Pearson, B.S., assistant chief of the Dairy Division of the Department of Agriculture, has already attracted much attention not only from those directly engaged in the dairy industry, but from many others whose alliance to these interests is less direct, but none the less important. The recent compilation and publication of *National and State Dairy Laws*, showing the progress, present status, and future needs in this direction, any of the greatest interest and importance alike to the dairyman and veterinarian. The salient points of all the laws of the several States are pointed out, and the most salutary provisions in the successful maintenance of this work are readily recognized. Those seeking legislation in these lines of work will find a useful guide in this report. The work of the author of this compilation—in the care and production of milk, the suggested lines of mutual work of boards of health, and those engaged in the dairy industry, and in many other aspects of this field of work—shows a deep interest, a broad conception of the situation; and what may be accomplished in a practical way on a commercial basis is never lost sight of. A similar compilation along the lines of an abstract of the various State laws relative to the control and eradication of contagious and infectious diseases of livestock would be very much appreciated.

